# Case report: Exploring efficacy of tofacitinib in modulating interferon response in five case of anti-MDA5+ dermatomyositis with interstitial lung disease

**DOI:** 10.3389/fimmu.2025.1515602

**Published:** 2025-02-04

**Authors:** Jie Zhao, Yan Bao, Ying Gao, Keliang Xie

**Affiliations:** Department of Critical Care Unit, Tianjin Medical University General Hospital, Tianjin, China

**Keywords:** MDA5-DM, RP-ILD, tofacitinib, interferon, cytokine

## Abstract

A case report highlights the challenges faced in managing a 66-year-old Chinese woman diagnosed with anti-MDA5 antibody-positive dermatomyositis (MDA5-DM) complicated by rapidly progressive interstitial lung disease (RP-ILD). Despite aggressive therapeutic interventions, her condition rapidly deteriorated, emphasizing the severity and devastating nature of this subtype of DM. A salient feature of her clinical presentation was the marked elevation of interferon (IFN)-γ and IFN-α levels, underscoring the pivotal role that IFNs play in driving the pathogenesis and progression of MDA5-DM-related RP-ILD. In an attempt to stem the relentless progression, tofacitinib, a Janus kinase (JAK) inhibitor, was applied into her treatment regimen. This therapeutic intervention led to a transient decrease in IFN-related cytokines, offering a glimpse of hope that JAK inhibition could modulate the exaggerated IFN response implicated in the disease. Other four similar cases underscore the critical importance of early and aggressive intervention in MDA5-DM patients, as well as the potential therapeutic avenues involving IFN blockers by JAK inhibitors. Urgent and well-designed clinical trials are imperative to unravel the intricate interplay between RP-ILD and the IFN signature in MDA5-DM, and to evaluate novel therapeutic targets that promise long-term efficacy and safety.

## Introduction

Anti-melanoma differentiation-associated protein 5 antibody-positive dermatomyositis (MDA5-DM) represents a distinct clinical entity within dermatomyositis spectrum, distinguished by its seropositivity to MDA5 autoantigen, cutaneous ulceration, rapidly progressive interstitial lung disease (RP-ILD), and minimal to absent muscular involvement. Notably, RP-ILD associated with MDA5-DM exhibits poor responsiveness to current therapeutic modalities, thereby becoming the primary cause of mortality in this subgroup (1).

Tofacitinib, a Janus kinase (JAK) inhibitor, has emerged as a promising salvage therapy for patients with high-risk ADM-ILD who have failed to respond to conventional treatments (2, 3). Accumulating evidence highlights the pivotal role of MDA5 in activating the JAK/Signal Transducer and Activator of Transcription (STAT) pathway, ultimately leading to the secretion of interferon (IFN) family cytokines (4). In this context, we report five cases of anti-MDA5-positive DM complicated by refractory RP-ILD, which were unresponsive to standard combined immunosuppressive therapies. Remarkably, these patients exhibited a diverse pattern of IFN-related cytokine alterations subsequent to tofacitinib administration.

## Case reports

### Case 1

**Figure 1:**
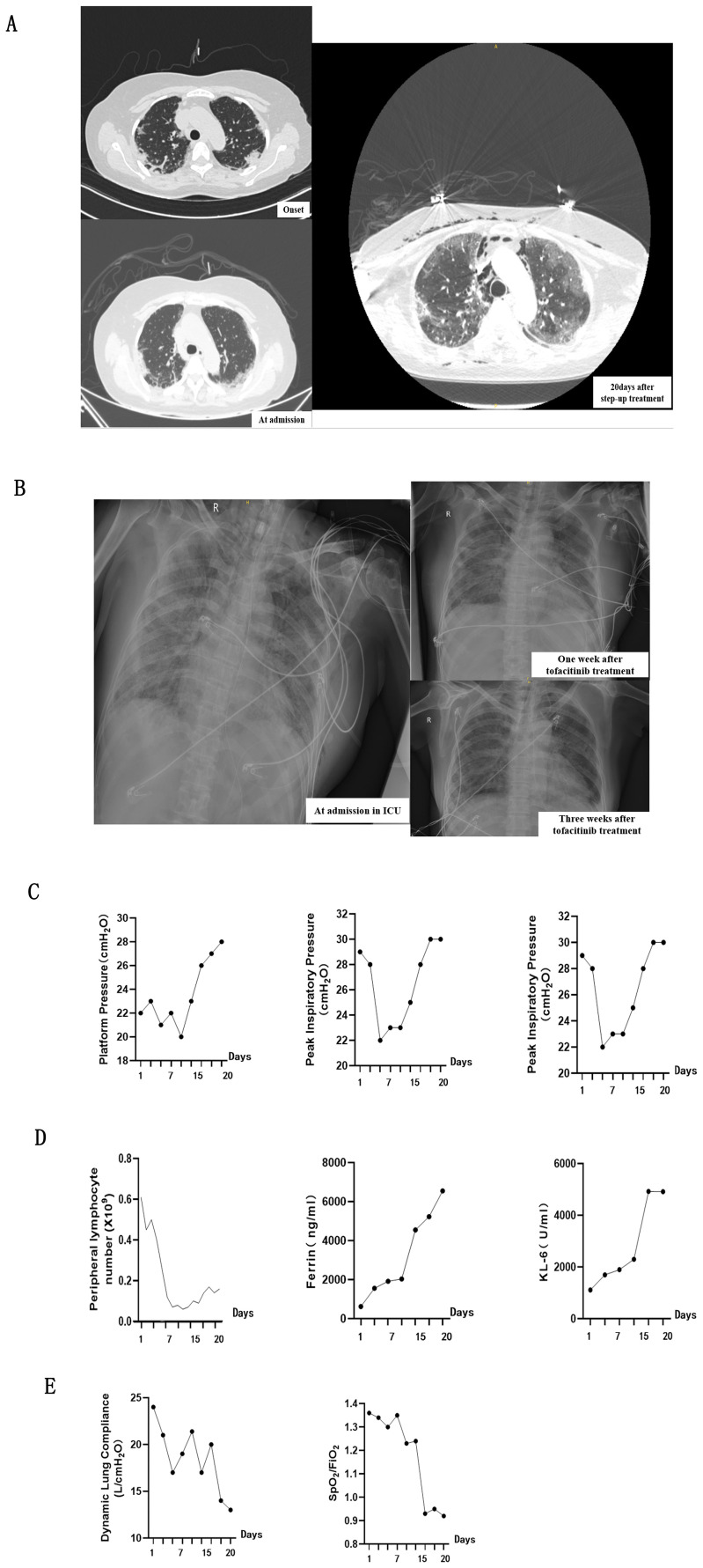
Comprehensive Assessment of clinical findings while the patient was receiving Tofacitinib treatment. **(A, B)** showcases chest imaging findings throughout entire treatment, including before and after Tofacitinib administration. **(A)** panel presents CT scans of the chest (lung window) before Tofacitinib treatment captured at three critical stages: initially upon admission to the Rheumatology Department, marking the onset of the disease, and again 20 days after the commencement of a step-up treatment regimen. **(B)** panel shows plain X-rays of the lungs after Tofacitinib treatment, captured at admission to the ICU, and one week and three weeks after the administration of tofacitinib, providing a temporal view of chest imaging findings from disease onset to the end of treatment. **(C)** presents a series of respiratory mechanics measurements conducted during the period of mechanical ventilation, specifically while the patient was receiving Tofacitinib at a dose of 5 mg twice daily for 20 days. **(D)** illustrates the serum levels of several key biomarkers throughout the treatment period, including peripheral blood lymphocytes, ferritin, and Krebs von den Lungen-6 (KL-6). **(E)** Decreased static lung compliance (CstatL) and pulse oximetry-measured oxygen saturation index (SpO_2_/FiO_2_) demonstrated the aggravation of interstitial lung injury.

A 66-year-old Chinese woman presented with non-productive intermittent dry cough and progressive dyspnea in the first 3 weeks. Then, she appeared DM-related skin rashes, including bilateral periorbital heliotrope rash and Gottron sign on the joints of the elbows, in the next one week. Chest high-resolution Computed Tomography (HRCT) demonstrated interlobular septal thickening and subpleural ground-glass opacities (**Figure 1A**). Laboratory examination found increased serum anti-MDA5 antibody titer(328.76 for normal range 200-250), with positive antinuclear antibody at titer of 1:80. She was referred to Department of Rheumatology one months after her symptom onset and was diagnosed with MDA5-DM.

**Figure 2:**
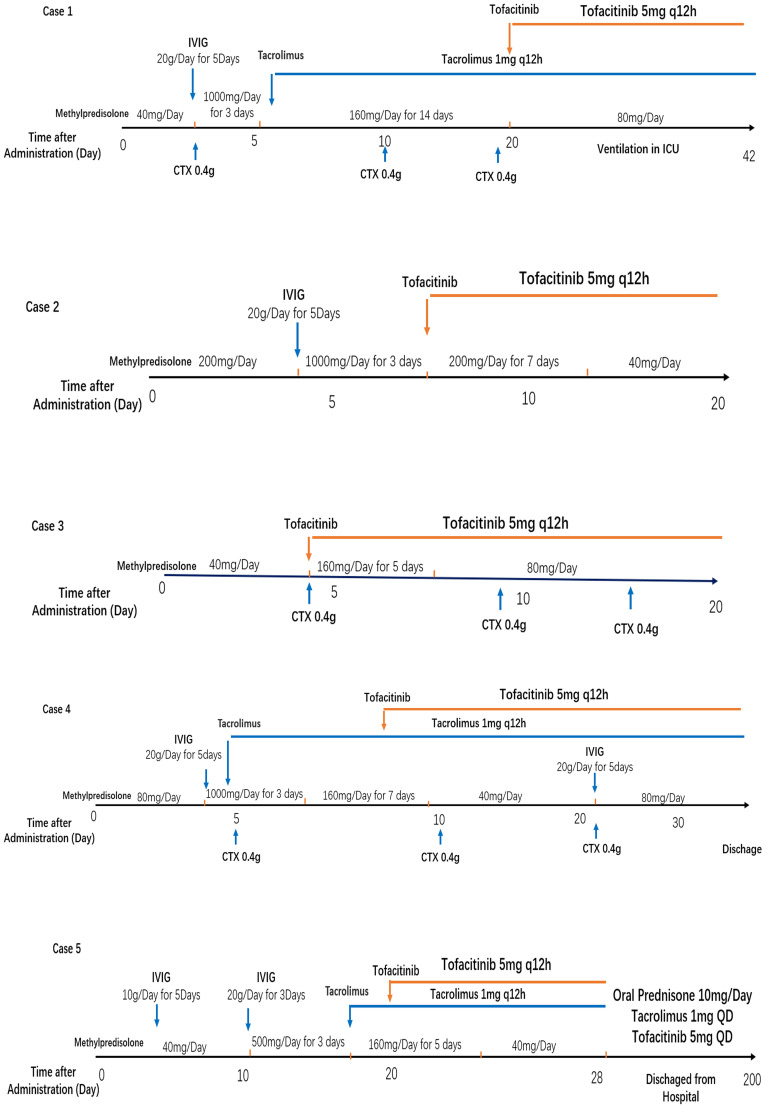
Treatment strategies for five enrolled MDA5-DM with rapidly progressive ILD patients. IVIG, intravenous immunoglobulin; CTX, cyclophosphamide.

A step-up treatment regimen was initiated immediately for the rapid progressive interstitial lung disease (RP-ILD) (**Figure 1A**), with a combination of high-dose methylprednisolone equivalent of 2 mg/kg/day (1000mg/day) for 3days, intravenous immunoglobulin 20 g/d for 5 days, intravenous cyclophosphamide at a dosage of 0.4 grams once weekly (QW), and oral tacrolimus 2mg/day. The patient was received high-dose intravenous methylprednisolone (160 mg/d for 2 weeks) in the following 20days of treatment (**Figure 2**). Her skin lesion and fever disappeared. However, chest HRCT showed a rapid progression of interstitial lesions (**Figure 1A**), and she required invasive ventilation for worsening hypoxemia after transferred to intensive care unit (ICU). On ICU admission, we decided to treat the patient with tofacitinib(at a dose of 5 mg twice daily) as her salvage therapy after she failed former combination therapy.

After administration to ICU, multiple indicators were conducted at the start of treatment and subsequently tested every 3-5 days. In the initial stage of tofacitinib therapy, a transient improvement in lung parenchymal translucency was observed on chest radiograph (**Figure 1B**). The improvement in respiratory effort markers, including plateau pressure (Pplat), peak inspiratory pressure (Ppeak), and driving pressure, occured simultaneously (**Figure 1C**). These changes suggested a potential therapeutic benefit of JAK inhibition in ameliorating pulmonary involvement. However, approximately three weeks after initial tofacitinib treatment, interstitial lung injury unexpectedly progressed despite these promising initial signs. Both chest radiograph and above respiratory parameters underwent deterioration (**Figures 1B, C**), accompanied by a series of changes in laboratory indicators. Increasement of serum ferritin levels and Krebs von den Lungen-6 (KL-6) levels, as well as a marked decrease in peripheral blood lymphocyte count (**Figure 1D**), were parallelled with the aggravation of interstitial lung injury. In line with these deterioration, decreased static lung compliance (CstatL) and pulse oximetry-measured oxygen saturation index (SpO_2_/FiO_2_) were accompanied during the treatment period (**Figure 1E**).

**Figure 3:**
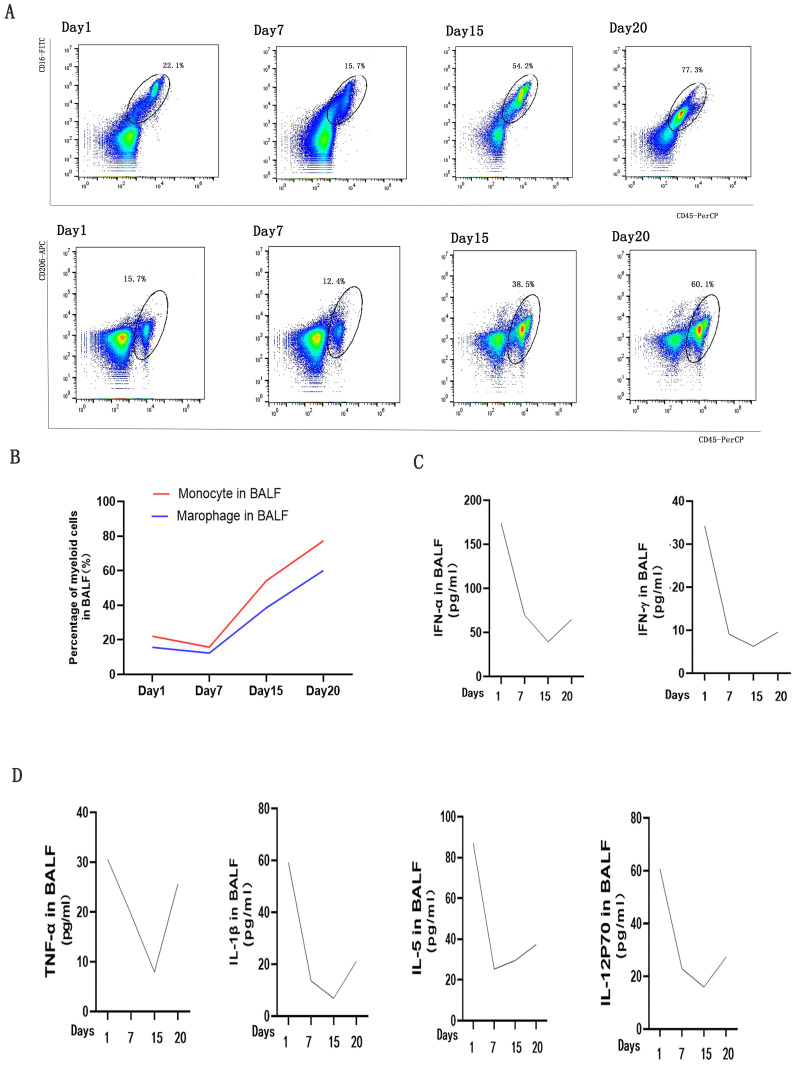
Analysis of Myeloid Cells and Cytokine/Chemokine Levels in bronchoalveolar lavage fluids (BALFs) during Tofacitinib treatment. **(A)** presents an analysis of myeloid cells in BALFs across intubation time points at day 1, day 7, day 15, and day 20. Monocyte was defined as CD45+CD16+(a), and macrophage was defined as CD45+CD206+ lymphocytes by flow cytometry analysis **(B)**. Changes of IFN signaling related cytokines **(C)** and cytokines/chemokines **(D)** in supernatants of BALFs were measured by cytometric bead array at the same time points.

Reports have documented the pivotal role of myeloid cells in triggering cytokine storms during the progression of RP-ILD worsens (5, 6). In the initial period of tofacitinib therapy, we observed percentages of CD45^+^CD16^+^ monocyte and CD45^+^CD206^+^ macrophage subsets(methods seen in Supplementary) maintaining stability in BALF (**Figure 3A**). However, as dyspnea progression, their accumulation accelerated (**Figure 3B**). Comprehensively, recent research has emphasized interferon(IFN) signaling pathways, including cytokines like IFN-γ and IFN-α (7), as key regulators of monocytes and macrophages in pulmonary disorders (8). As we expected, secretions of IFN-γ and IFN-α in BLAF reduced in the initial period of tofacitinib therapy, subsequently increased as interstitial lung disease progressed to uncontrollable levels afterwards (**Figure 3C**). Moreover, pervious study suggested that alveolar macrophage triggered the release of cytokines and chemokines in critical lung disease. We observed alterations in proinflammatory cytokines, particularly IL-1β and TNF-α, accompanying with monocyte/macrophage chemotaxis, including IL-5 and IL-12p70(methods seen in Supplementary). These immune biomarkers, which are associated with macrophage activity, demonstrated an initial transient decrease followed by a subsequent increase, similar with the accumulation of alveolar myeloid cells (**Figure 3D**). The comprehensive deep-immune atlas of RP-ILD suggest that pronounced myeloid-driven immune process and increased immune mediators are predominant immunological hallmarks of critical MDA5-DM patients.

Unfortunately, three weeks after initial tofacitinib treatment, she was complicated by worsening liver failure. Her respiratory status demonstrated continued decline despite ventilator support and she ultimately passed in the setting of pulseless electrical activity arrest due to hypoxemic respiratory failure.

### Cases series to estimate the role of tofacitinib in modulating Interferon response

From April 2022 to September 2023, our clinical investigation extended to another cohort of four patients diagnosed with MDA5-DM complicated by refractory RP-ILD (**Table 1**). The mean age of disease onset was 46 years (range 24–61 years), three females and one males were included. They all had significant increased serum anti-MDA5 antibody titer(range from 319.5-584.46), and three female patients were anti-Ro52-positive. Chest HRCT scans all revealed an organizing pneumonia pattern with subpleural consolidation, from subpleural opacities at the initial period to wide consolidation as ILD progression.

**Table 1:** Clinical features of patients treated with tofacitinib for refractory RP-ILD in anti-MDA5-positive dermatomyositis.

Characteristics	Case 2	Case 3	Case 4	Case 5
Age, years	24	40	60	61
Sex	Male	Female	Female	Female
MSA/MAA	Anti-MDA5-Ab^+^	Anti-MDA5-Ab^+^, antiRo52-Ab^+^	Anti-MDA5-Ab^+^, antiRo52-Ab^+^	Anti-MDA5-Ab^+^, antiRo52-Ab^+^
Initial treatment	MP, IVIG	MP, CTX	MP,IVIG,CTX, Tacrolimus	MP,IVIG, Tacrolimus
Symptoms	Rash	Rash, fever	Rash, dyspnea	Rash, fever
Tofacitinib administration time since symptom onset, weeks	2	1	2	3
On admission				
counts of peripheral blood lymphocytes, X10^9^/l	0.4	0.25	0.43	0.26
Ferritin, lg/l	1730.90	718.86	4731.97	4077.87
KL-6, lg/l	212	290	975	653
oxygenation index, mmHg	256	190	132	201
SpO_2_	97% with NC 5 l/min	94% with NC 5 l/min	87% with HFN 40 l/min	93% with NC 5 l/min
peripheral blood IFN-α, pg/ml	5.5	27.12	16.99	15.53
peripheral blood IFN-γ, pg/ml	3.12	31.89	9.08	8.72
Response to tocilizumab (after discharge)				
counts of peripheral blood lymphocytes, X10^9^/l	1.21	0.81	0.06	0.31
Ferritin, lg/l	101.44	107.41	6912.61	1692.32
KL-6, lg/l	negative	330	2486	289
oxygenation index, mmHg	287	310	87	187
SpO_2_	97% with NC 5 l/min	95% with NC 5 l/min	82% with HFN 40 l/min	90% with NC 5 l/min
peripheral blood IFN-α, pg/ml	1.3	1.33	50.05	9.24
peripheral blood IFN-γ, pg/ml	0.96	0.87	46.02	2.98
Duration of the combination therapy with tofacitinib, weeks	6	14	3	25
Prognosis	Alive	Alive	Treatment withdrawn	Alive

AA, ambient air; Abþ, antibody positive; FiO _2_, fraction of inspired oxygen; HFN, high-flow nasal oxygen therapy; MAA, myositis-associated antibody; MP, methylprednisolone; MSA, myositis specific antibody; NC, nasal cannula; SpO _2_, pulse oximeter oxygen saturation; IVIG, intravenous immunoglobulin.

**Figure 4:**
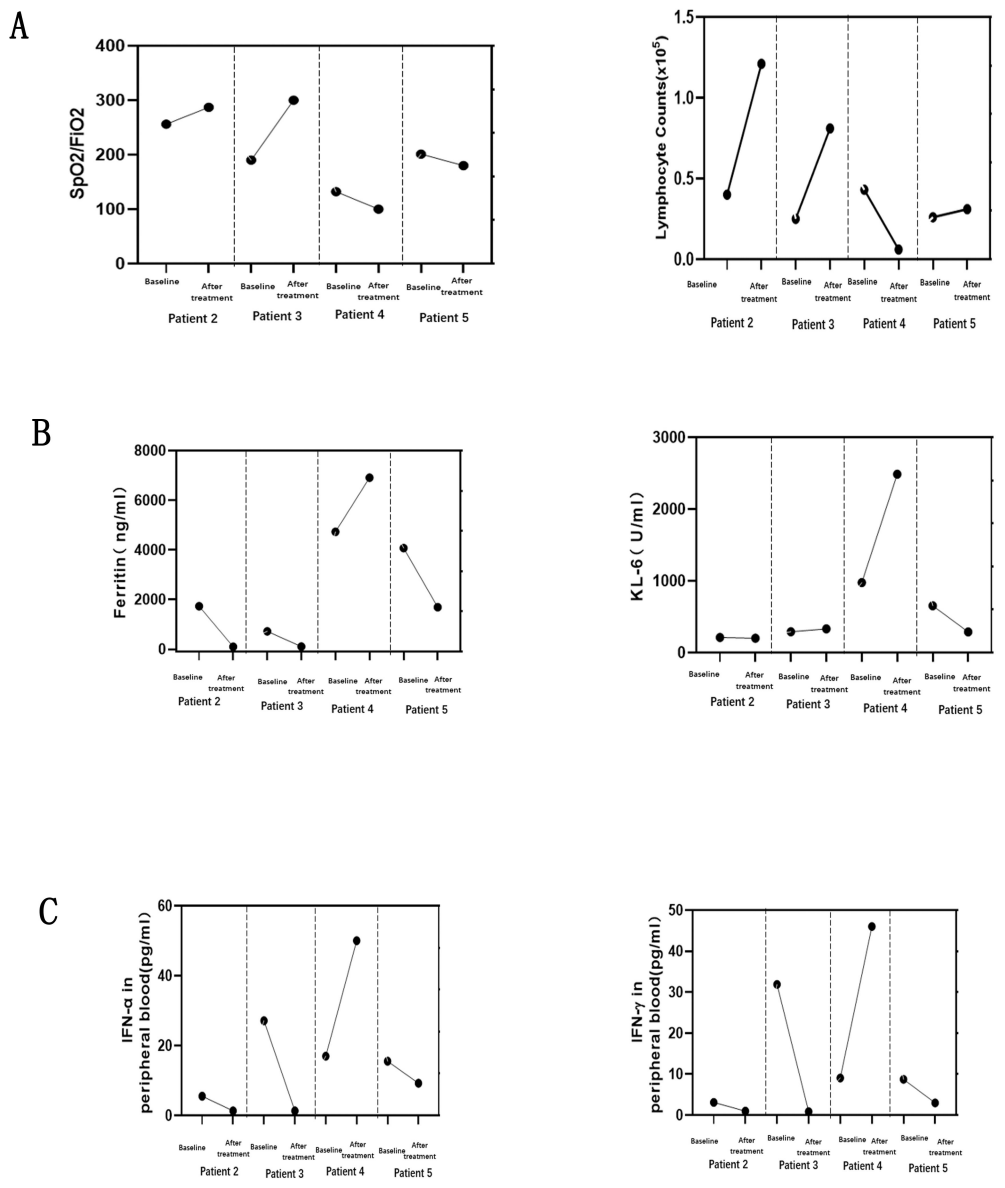
Changes in four MDA5-DM patients before and after treatment with tofacitinib. Interstitial lung injury demonstrated by static lung compliance (CstatL) and pulse oximetry-measured oxygen saturation index (SpO_2_/FiO_2_) **(A)**, with the serum levels of key biomarkers for ferritin, and Krebs von den Lungen-6 (KL-6) **(B)**. Changes of IFN signaling related cytokines **(C)** in peripheral blood were measured by cytometric bead array at the same time points.

Glucocorticoid is used as the first-line treatment in all four patients. The initial dose of methylprednisolone is dependent on the extent of disease severity. Among them, a high-dose methylprednisolone equivalent of 1–2 mg/kg/day(500-1000mg/day) of prednisone is administered in three patients with high disease activity, and a moderate dose (≤0.5 mg/kg/day of prednisone equivalent) of 160mg/day for case 3 with low disease activity. A combination of glucocorticoid with immunosuppressant therapy, including cyclophosphamide and tacrolimus, is also used in the initial treatment of three female patients as dyspnea progression. Meanwhile, intravenous immunoglobulin therapy has been established in three patients with high ILD activity. After all above triple combination therapy, JAK inhibition of oral tofacitinib(at a dose of 5 mg twice daily) were all administrated in these four refractory MDA5-DM patients (**Figure 2**). After approximate 20 days of treatment, case2 and case 3 patients achieved clinically significant improvement accompanied with increase in the absolute peripheral lymphocyte number and SpO_2_/FiO_2_ (**Figure 4A**). Biomarkers for lung damage, including ferritin and Krebs von den Lungen‐6 (KL‐6), also decreased during disease remission (**Figure 4B**). Case 5 patients attained limited effectiveness in maintaining stable levels of peripheral lymphocyte count, oxygen index and biomarkers in the initial treatments. However, case 5 patient showed major improvement after 6 months of oral triple combination therapy (**Figures 4A, B**). Only case 3 was worsened 20 days after treatment. Case 3 patient showed significant decrease in the absolute peripheral lymphocyte number and SpO_2_/FiO_2_, with elevation levels of serum ferritin KL‐6 (**Figures 4A, B**).Eventually, her family gave up on treatment and discharged from hospital.

The serum interferon(IFN) signaling pathways related cytokines, IFN-γ and IFN-α, at the baseline were higher in case 3 than those three alive patients (**Figure 4C**). The changes in IFN-γ and IFN-α were parallel to the deterioration of peripheral lymphocyte count, oxygen index and biomarkers in case 3 patient (**Figures 4A–C**). The levels of IFN-related cytokines showed a tight link to the progression of ILD from initial to post-treatment of tofacitinib. Specifically, all three alive patients exhibited a pronounced therapeutic response to tofacitinib. The significant reduction in IFN-mediated cytokines in three alive patients suggests that by targeting JAK pathways, tofacitinib effectively modulates the immune response, mitigating the proinflammatory environment associated with DM and potentially ameliorating interstitial lung injury.

## Discussion

MDA5+ dermatomyositis (DM) exhibits a poor prognosis, largely due to the common occurrence of rapidly progressive interstitial lung disease (RP-ILD), which critically influences disease progression and mortality, especially in the early and critical stages (9). Thus, identifying predictive markers for RP-ILD development and mortality is of utmost clinical significance. The rarity and high mortality rate of MDA5+ DM have limited the conduct of randomized controlled trials, leading to a lack of standardized treatment protocols. Current initial treatment approaches typically involve high-dose glucocorticoids, often combined with calcineurin inhibitors and cyclophosphamide. However, these treatment regimens are associated with a considerable short-term mortality rate, reaching as high as 50% (10).

Given this backdrop, Tofacitinib, a Janus kinase (JAK) inhibitor, has emerged as a promising therapeutic option for early-stage anti-MDA5+ ADM-related ILD. Clinical trials involving Tofacitinib in patients with MDA5-ILD have yielded remarkable results, showcasing improved survival rates, substantial enhancements in ferritin levels, pulmonary function tests, and HRCT imaging outcomes, all while maintaining a favorable safety profile with minimal adverse events (11, 12).

Several studies have reported poor prognostic factors in MDA5-DM (13–15). In this case series, all five patients had multiple risk factors (RP-ILD, lymphopenia, elevated ferritin). Thus, our experience should imply that the application of tofacitinib was effective for refractory MDA5-DM patients even with multiple risk factors. On the other hand, two fatal cases were already under severe dyspnea or even mechanical ventilation at the time of tofacitinib administration. It might be difficult to save a case whose pulmonary involvement has already severely progressed, even with any intensive therapies including tofacitinib.

The rationale for tofacitinib’s potential efficacy in managing RP-ILD complications in MDA5+ DM lies in the disease’s intricate relationship with the interferon (IFN) pathway (16). Our cluster analysis of cytokine profiles revealed significantly elevated levels of IFN-related cytokines (IFN-γ, IFN-α) in both serum and bronchoalveolar lavage fluid (BALF) samples from MDA5+ DM patients with RP-ILD. The MDA5 pattern recognition receptor triggers an abnormal overproduction of IFNs, which subsequently initiates a cascade of inflammatory responses and tissue damage (17). This exaggerated IFN response, mirrored in both blood and lung lavage fluids, resembles a surge of circulating cytokines, chemokines, and activated macrophage-related proteins, contributing to the release of proinflammatory factors and thereby accelerating disease progression (18).

Given this, interferon blockers, as a mechanism-based treatment approach, aimed at reducing inflammation throughout the body and organs, may represent the future direction in the treatment of MDA5+ DM-related ILD patients. Furthermore, it has been confirmed that treatment with tofacitinib, in combination with glucocorticoids, can significantly improve the survival rates of early-stage RP-ILD patients compared to those treated with conventional immunosuppressants (19).

This is a small case series without a control group and therefore our study suffers from several important limitations. First, results of this study may have been affected by the objective evaluation of different physicians. Second, owing to the small number of patients included in this study and lack of a control group, caution is required in the interpretation of the results. Last, although our analysis indicated that RP-ILD in MDA5-DM patients were associated with myeloid-driven immune process and IFN-related immune mediators, validation studies using cell line and animal models are needed in future.

Insights into the interplay between RP-ILD and the IFN signature may provide novel avenues for understanding the immunopathogenesis of MDA5+ DM and identifying potential therapeutic targets. Our case report suggested that tofacitinib might be a salvage therapy in treating fatal RP-ILD with MDA-5 DM. To ascertain the prognostic significance of IFN-related cytokine levels, further research is imperative. Additionally, well-structured, multicenter, randomized controlled trials are indispensable for comprehensively evaluating the long-term effectiveness and tolerability of tofacitinib in the management of MDA5-DM-associated ILD.

## Data Availability

The raw data supporting the conclusions of this article will be made available by the authors, without undue reservation.
