# Supplementary material for: Case report: Exploring efficacy of tofacitinib in modulating interferon response in five case of anti-MDA5+ dermatomyositis with interstitial lung disease
Source: Front Immunol. 2025 Feb 4;16:1515602. doi: 10.3389/fimmu.2025.1515602 (PMC11832562; doi:10.3389/fimmu.2025.1515602)
Supplement: Supplementary file 1 [file Presentation1.pdf]

Supplementary for methods

Sample collection

BALF was collected and preserved undiluted from all intubated patients during a bronchoscopy performed. The bronchoscope was wedged in the segments of interest based on available chest imaging or intra-procedure observations. Aliquots of normal saline (20 mL each) were instilled through the bronchoscope within the selected bronchopulmonary segment. After each aliquot was instilled, saline was retrieved using a negative suction pressure. Approximately 20 ml of BALF was obtained and placed on ice. After passage of BALF through a 100-μm nylon cell strainer to remove clumps and debris, the supernatant was centrifuged. Supernatant was stored at −80°C and BAL cell pellets were collected in phosphate buffered saline solution(PBS) for flow cytometry.

Flow cytometry

The immunophenotyping procedure was performed less than 6 hours after sample collection. Approximately 1 × 106 cells per sample were surface stained for 30 min in the dark with the following fluorochrome-labeled antibodies: PE-conjugated anti-CD16, Percp-conjugated anti-CD45, APC-conjugated anti-CD206 (eBioscience). After washing cells by adding 1 mL PBS and centrifuging for 5 min at 400 g, the cells were resuspended in 400 μL PBS and examined by BD FACScantoTM II flow cytometer using the FACSDiva v. 6.1 software.

Cytokine analysis

Cytokine measurements were assessed by multiplex analysis performed on fluorescently labeled magnetic microsphere beads using the LegendPlex Human Inflammation Panel 1 (13-plex) (740809, BioLegend), according to the manufacturer's instructions. The concentrations of 13 biomarkers in whole samples, including inflammatory markers and cytokines/chemokines (interleukin (IL)-1β, IL-2, IL-6, IL-8, IL-10, IL-12 (p40), IL-12 (p70), IL-23, IL-17, interferon (IFN)-γ, IFN-α, tumor necrosis factor (TNF)-α, IL-23), were quantified and expressed in terms of fluorescence intensities and concentrations (pg/mL). The calibration curve was created separately for each analyte. Briefly, pre-stored supernatant was taken. Then, 25 μl of sonicated beads, 25 μl of sample, and 25 μl of detection antibodies were mixed and placed on a shaker at 500 r.p.m. for 2 h at room temperature. Subsequently, 25 μl of SA-PE was added directly to each tube. The tubes were placed on a shaker at 500 r.p.m. for 30 min. The data were obtained by flow cytometry and were analyzed using LEGENDplex v.10.0 (VigeneTech).
